# Intentional and Unintentional Medication Non-Adherence in Psoriasis: The Role of Patients’ Medication Beliefs and Habit Strength

**DOI:** 10.1016/j.jid.2017.11.015

**Published:** 2018-04

**Authors:** Rachael J. Thorneloe, Christopher E.M. Griffiths, Richard Emsley, Darren M. Ashcroft, Lis Cordingley, British Association of Dermatologists Biologic Interventions Register, Jonathan Barker, Marilyn Benham, David Burden, Ian Evans, Christopher Griffiths, Sagair Hussain, Brian Kirby, Linda Lawson, Kayleigh Mason, Kathleen McElhone, Ruth Murphy, Anthony Ormerod, Caroline Owen, Nick Reynolds, Catherine Smith, Richard Warren, Psoriasis Stratification to Optimise Relevant Therapy Study Groups, Jonathan Barker, Michael Barnes, David Burden, Richard Emsley, Christopher Griffiths, Katherine Payne, Nick Reynolds, Samantha Ryder, Catherine Smith, Deborah Stocken, Richard Warren

**Affiliations:** 1 Division of Pharmacy and Optometry, School of Health Sciences, Faculty of Biology, Medicine and Health, University of Manchester, Manchester, UK; 2 Centre for Dermatology Research, Manchester Academic Health Science Centre and NIHR Manchester Biomedical Research Centre, University of Manchester, Manchester, UK; 3 Salford Royal Hospital NHS Foundation Trust, Salford, UK; 4 Division of Musculoskeletal and Dermatological Sciences, Manchester Academic Health Science Centre and NIHR Manchester Biomedical Research Centre, University of Manchester, Manchester, UK; 5 Centre for Biostatistics, School of Health Sciences, University of Manchester, Manchester Academic Health Science Centre, Manchester, UK; 6 Centre for Pharmacoepidemiology and Drug Safety, Manchester Academic Health Science Centre, University of Manchester, Manchester, UK

**Keywords:** BADBIR, British Association of Dermatologists Biologic Interventions Register, CI, confidence interval, LPA, latent profile analysis, OR, odds ratio

## Abstract

Medication non-adherence is a missed opportunity for therapeutic benefit. We assessed “real-world” levels of self-reported non-adherence to conventional and biologic systemic therapies used for psoriasis and evaluated psychological and biomedical factors associated with non-adherence using multivariable analyses. Latent profile analysis was used to investigate whether patients can be categorized into groups with similar medication beliefs. Latent profile analysis categorizes individuals with similar profiles on a set of continuous variables into discrete groups represented by a categorical latent variable. Eight hundred and eleven patients enrolled in the British Association of Dermatologists Biologic Interventions Register were included. Six hundred and seventeen patients were using a self-administered systemic therapy; 22.4% were classified as “non-adherent” (12% intentionally and 10.9% unintentionally). Patients using an oral conventional systemic agent were more likely to be non-adherent compared to those using etanercept or adalimumab (29.2% vs. 16.4%; *P* ≤ 0.001). Latent profile analysis supported a three-group model; all groups held strong beliefs about their need for systemic therapy but differed in levels of medication concerns. Group 1 (26.4% of the sample) reported the strongest concerns, followed by Group 2 (61%), with Group 3 (12.6%) reporting the weakest concerns. Group 1 membership was associated with intentional non-adherence (odds ratio = 2.27, 95% confidence interval = 1.16−4.47) and weaker medication-taking routine or habit strength was associated with unintentional non-adherence (odds ratio = 0.92, 95% confidence interval = 0.89−0.96). Medication beliefs and habit strength are modifiable targets for strategies to improve adherence in psoriasis.

## Introduction

Conventional systemic and biologic therapies are highly effective in the treatment of moderate to severe psoriasis; however, treatment effectiveness is much lower in routine clinical practice than in clinical trials (Gelfand et al., 2012, Iskandar et al., 2017b). Patients need to adhere to their medication, yet approximately half of medications are not taken as prescribed (Nieuwlaat et al., 2014). There is a lack of high-quality data on levels of adherence to systemic therapies outside of clinical trials, and the factors influencing adherence are largely unknown (Thorneloe et al., 2013).

Non-adherence can be intentional, where patients make a deliberate decision not to follow the prescribed medication regimen, such as altering the dose, timing, or frequency of their systemic therapy. In other disease groups, patients’ beliefs about their condition and medication are important drivers of non-adherence (Horne et al., 2013, Leventhal et al., 2016). Patients’ evaluations of personal need for medication for current and future health (necessity beliefs) and concerns about potential negative effects from treatment (concern beliefs) are important medication beliefs that influence intentional non-adherence, as posited by the extended Common-Sense Self-Regulatory Model of illness (Leventhal et al., 1992) and treatment (Horne and Weinman, 1999, Horne and Weinman, 2002).

Non-adherence can also be unintentional, such as forgetting to use the medication. The strength of the patient’s routine, or habit, for taking their medication has been shown to influence unintentional non-adherence, especially when the patient has been using their treatment for an extended period of time (Danner et al., 2008, Leventhal et al., 2016, Phillips et al., 2013, Phillips et al., 2016).

The aim of the Psoriasis Stratification to Optimise Relevant Therapy consortium is to better understand the determinants of treatment response to biologic therapies in psoriasis in order to develop a clinical algorithm to help stratify people to the most appropriate first-line biologic therapy and overcome trial-and-error prescribing (Griffiths et al., 2015). In order to develop this stratified approach and optimize treatment effectiveness, it is necessary to examine non-adherence and understand its “modifiable” risk factors. The iMAP (Investigating Medication Adherence in Psoriasis) study is a large national multi-site study collecting biomedical and psychological data from patients prescribed systemic therapies at 35 dermatology centers in England. All patients are enrolled in the British Association of Dermatologists Biologic Interventions Register (BADBIR), a pharmacovigilance register representing a “real-world” cohort of psoriasis patients using systemic therapies (Burden et al., 2012, Iskandar et al., 2017a).

The objectives of this study were to: (i) assess real-world levels of intentional and unintentional non-adherence to conventional systemic and biologic therapies used for psoriasis; (ii) assess whether patients can be categorized into groups with similar medication beliefs and; (iii) examine whether psychological and biomedical factors are associated with non-adherence to systemic therapies. To identify the optimal number of medication belief groups, we used latent profile analysis (LPA), which categorizes individuals with similar profiles on a set of continuous variables (medication beliefs) into discrete groups or latent classes, represented by a categorical latent variable (medication belief group).

## Results

### Demographic, clinical, and psychological data

A total of 811 patients using a systemic therapy for the treatment of psoriasis were included in the study (Table 1). In the biologic cohort (64.7%, n = 525), the most common prescribed biologic was adalimumab (52.5%; n = 258), followed by ustekinumab (32.6%; n = 160) and etanercept (14.9%; n = 73). In total, 75.4% (n = 352) were prescribed their first biologic therapy (biologic naïve) and 7% (n = 36) were using a concomitant systemic therapy. In the conventional systemic cohort (35.3%; n = 286), methotrexate was the most common (52%; n = 141), followed by cyclosporine (21.8%; n = 59), acitretin (19.2%; n = 52), and fumaric acid esters (7%; n = 19).

**Table 1:** Demographic, clinical, and psychological data at baseline (n = 811)

Characteristics	Total Sample (n = 811)	Conventional Cohort^1^ (35.3%, n = 286)	Biologic Cohort^2^ (64.7%, n = 525)	*P-*Value^3^
Demographic				
Age, y, mean ± SD	48.1 ± 13.1	48.4 ± 13.7	47.9 ± 12.8	0.599
n	720	254	466	
Male, % (n)	57.1 (411)	49.2 (125)	61.4 (286)	**0.002**
n	720	254	466	
Disease				
Age of onset, y, mean ± SD	25.4 ± 15.1	27.8 ± 16.8	24.1 ± 13.9	**0.001**
n	716	252	464	
Disease duration, y, mean ± SD	23.3 ± 13.8	21.3 ± 14.8	24.3 ± 13.0	**0.004**
n	718	253	465	
PASI at the start of treatment, mean ± SD	14.3 ± 7.0	14.8 ± 7.5	14.0 ± 6.7	0.164
n	693	245	448	
DLQI at the start of treatment, mean ± SD	13.7 ± 7.9	15.6 ± 6.3	12.6 ± 8.5	**≤0.001**
n	684	244	440	
Comorbidities				
Presence of ≥1 comorbidity,^4^ % (n)	65.2 (471)	62 (158)	67 (313)	0.172
n	722	255	467	
Inflammatory arthritis, % (n)	17.3 (124)	9.5 (24)	21.5 (100)	**≤0.001**
n	718	253	465	
Treatment				
Treatment duration, mo, mean ± SD	11.4 ± 13.5	7.0 ± 11.1	13.9 ± 14.1	**≤0.001**
n	744	265	479	
Psychological distress (HADS^5^)				
Anxiety, mean ± SD	6.7 ± 4.3	6.9 ± 4.4	6.5 ± 4.3	0.168
n	794	281	513	
Depression, mean ± SD	4.8 ± 4.0	5.3 ± 4.2	4.5 ± 3.9	**0.010**
n	796	280	516	
Illness beliefs and emotional response toward psoriasis (IPQ-R^6^)				
Illness identity^7^ (symptoms), mean ± SD	4.2 ± 2.8	4.1 ± 2.6	4.3 ± 2.9	0.420
n	784	275	509	
Timeline acute/chronic, mean ± SD	4.2 ± 0.6	4.2 ± 0.6	4.3 ± 0.6	**0.020**
n	796	279	517	
Timeline cyclical, mean ± SD	3.3 ± 0.7	3.3 ± 0.7	3.2 ± 0.7	0.080
n	803	284	519	
Consequences, mean ± SD	3.7 ± 0.7	3.6 ± 0.7	3.7 ± 0.7	0.113
n	804	286	518	
Personal controllability, mean ± SD	3.0 ± 0.7	3.0 ± 0.7	3.0 ± 0.8	0.365
n	800	281	519	
Treatment controllability, mean ± SD	3.7 ± 0.6	3.6 ± 0.5	3.8 ± 0.6	**≤0.001**
n	792	282	510	
Coherence (understanding), mean ± SD	3.4 ± 0.9	3.2 ± 0.9	3.5 ± 0.9	**≤0.001**
n	800	281	519	
Emotional response, mean ± SD	3.4 ± 0.8	3.5 ± 0.8	3.4 ± 0.9	0.702
n	802	284	518	
Medication beliefs (BMQ^6^)				
Specific necessity, mean ± SD	3.7 ± 0.8	3.5 ± 0.8	3.9 ± 0.8	**≤0.001**
n	795	281	514	
Specific concerns, mean ± SD	2.5 ± 0.7	2.7 ± 0.7	2.3 ± 0.6	**≤0.001**
n	794	280	514	
General overuse, mean ± SD	2.7 ± 0.7	2.7 ± 0.7	2.7 ± 0.7	0.287
n	804	286	518	
General harmfulness, mean ± SD	2.3 ± 0.6	2.3 ± 0.6	2.2 ± 0.6	**0.029**
n	802	286	516	

Abbreviations: BMQ, Beliefs about Medicines Questionnaire; DLQI, Dermatology Life Quality Index; HADS, Hospital Anxiety and Depression Scale; IPQ-R, Revised Illness Perception Questionnaire; PASI, Psoriasis Area and Severity Index; SD, standard deviation.

1 Includes methotrexate, cyclosporine, acitretin, and fumaric acid esters.

2 Includes adalimumab, ustekinumab, and etanercept.

3 The *P*-value tests for significant differences between the conventional systemic and biologic cohorts using *t*-tests and χ^2^ tests. Boldface indicates *P* < 0.05.

4 Includes any of (excluding psoriatic arthritis) hypertension, angina, ischemic heart disease, stroke, epilepsy, asthma, chronic obstructive pulmonary disease, peptic ulcer, renal disease, hepatic disease, tuberculosis, demyelinating disease, diabetes, impaired glucose tolerance, depression, non-skin cancer, immunodeficiency syndrome, thyroid disease, other.

5 The possible score range for HADS anxiety and HADS depression is 0−21.

6 The possible score range for all IPQ-R and BMQ items (excluding illness identity) is 1−5.

7 The possible score range for illness identity is 0−17.

Patients in the biologic cohort were significantly more likely to be male; have younger age of onset of psoriasis; longer duration of disease; more likely to have a diagnosis of inflammatory arthritis; report lower Dermatology Life Quality Index scores at the start of their systemic therapy; have longer duration of systemic therapy; hold stronger beliefs in the chronicity of their psoriasis; stronger beliefs that their systemic therapy is necessary and helps to control and manage their symptoms; weaker concerns about their systemic therapy and medicines in general; greater perceived understanding of psoriasis and its causes and triggers (coherence); and report fewer symptoms of depression compared to those in the conventional cohort.

### Non-adherence to systemic therapies and medication-taking habit/routine strength

Only 7.3% (n *=* 11) of patients using ustekinumab were classified as overall non-adherent. Ustekinumab is predominately nurse-administered in the United Kingdom and this value may reflect the contrast between regular patient self-administered and 12-weekly nurse-administered injection in most centers.

**Figure 1:**
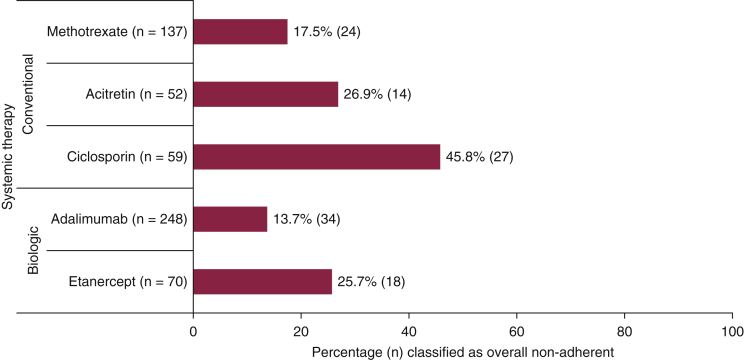
**Proportion of patients classified as overall non-adherent within the oral conventional systemic and self-administered biologic cohorts.** Fumaric acid esters were excluded due to small sample size (7%, n = 19).

Table 2 presents the proportion of patients classified as non-adherent to self-administered systemic therapies; those on ustekinumab (n = 160) and those with missing biologic treatment type (n = 34) were excluded. A significant proportion of patients using self-administered oral conventional systemic or subcutaneous biologic therapies (etanercept, adalimumab) were classified as non-adherent (22.4%; n = 134); 12% (n = 72) were classified as intentionally and 10.9% (n = 66) were classified as unintentionally non-adherent (Table 2). A higher proportion of patients prescribed an oral conventional systemic were classified as non-adherent compared to those prescribed etanercept or adalimumab (29.2% [n = 82] vs. 16.4% [n = 52]; p ≤ 0.001). Figure 1 presents the proportion of patients classified as overall non-adherent within the oral conventional systemic and self-administered biologic cohorts. Fumaric acid esters were excluded from Figure 1 due to small sample size (7%; n = 19). As shown in Figure 1, a higher proportion of patients prescribed etanercept were classified as non-adherent compared with adalimumab. A higher proportion of patients prescribed cyclosporine were classified as non-adherent compared with acitretin or methotrexate.

**Table 2:** Proportion of patients classified as non-adherent to self-administered systemic therapies (n = 617)

Adherence	Total Sample	Conventional Cohort^1^	Biologic Cohort^2^	*P-*value^3^
Overall non-adherent, % (n)	22.4 (134)	29.2 (82)	16.4 (52)	**≤0.001**
n	599	281	318	
Intentionally non-adherent, % (n)	12 (72)	15.3 (43)	9.1 (29)	**0.020**
n	599	281	318	
Unintentionally non-adherent, % (n)	10.9 (66)	14.5 (41)	7.7 (25)	**0.007**
n	605	282	323	

1 Includes methotrexate, cyclosporine, acitretin and fumaric acid esters.

2 Includes adalimumab and etanercept.

3 *P*-value tests for significant differences between the conventional systemic and biologic cohorts using χ^2^ tests. Boldface indicates *P* ≤ 0.05.

Using the Self-Report Habit Index (Verplanken and Orbell, 2003), patients using a self-administered systemic therapy reported strong routine, or habit, for taking their systemic therapy, with a mean ± standard deviation score of 41.5 ± 9.7 out of a possible score of 60. A high proportion of the sample agreed or strongly agreed that taking their systemic therapy is something they do “frequently” (91.2%, n = 542), “automatically” (85.2%, n = 509), and “as part of their routine” (83.7%, n = 498).

### Medication belief groups

**Figure 2:**
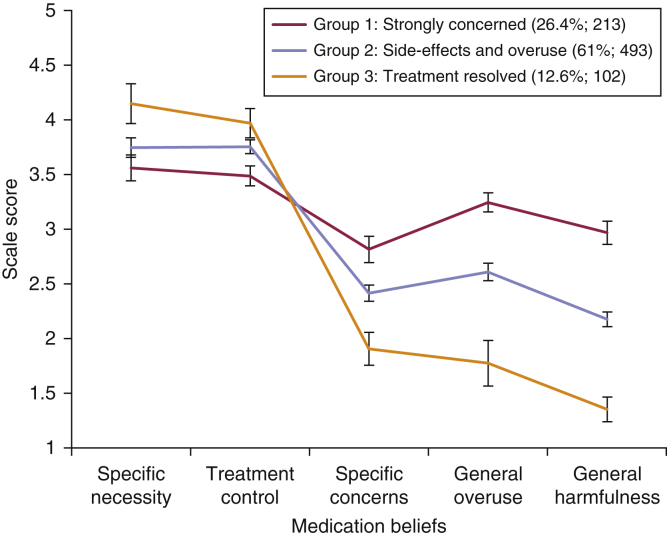
**Sample means (95% confidence intervals) of medication beliefs for the three-class solution.**

The optimal number of medication belief groups was identified using LPA. The model fit statistics for 1- to 5-class solutions indicated a 3-class solution was optimal (Supplementary Table S1 online); patients can be classified into three groups characterized by distinct medication beliefs. Sample means (95% confidence intervals [CIs]) of medication beliefs for the 3-class solution are presented in Figure 2.

Each of the three medication belief groups perceived a strong need for their systemic therapy (specific necessity) and believed that their therapy was effective in managing their psoriasis symptoms (treatment controllability), but differed in their level of medication concerns.

Group 1 (26.4%; n = 213) was labeled “strongly concerned.” Patients in this group reported the strongest concerns about their systemic therapy, these included potential long-term adverse effects and future medication dependency. Patients believed they lacked understanding about their systemic therapy. Those in Group 1 also reported the strongest concerns about the overuse of medicines in general: patients believed that doctors use too many, and place too much trust on medicines, and that doctors would prescribe fewer medicines if they had more time with patients. Patients also reported the strongest concerns about the harmfulness of medicines in general, including strong beliefs that people who take medicines should stop their treatment for a while every now and again, that most medicines “are addictive,” “are poisons,” and “do more harm than good.”

Group 2 (61%, n = 493) was labeled “side effects and overuse.” Although patients reported weaker medication concerns than Group 1, they still expressed strong concerns about potential long-term adverse effects from their systemic therapy and concerns about the general overuse of medicines. Group 3 (12.6%, n = 102) was labeled “treatment resolved”; patients reported far lower ratings for medication concerns compared to Group 1 and 2.

The proportion of patients classified into the three medication belief groups significantly differed by treatment cohort (*P* ≤ 0.001), with a higher proportion of patients in the biologic cohort classified into Group 3 (15.5%; n = 81) compared with the conventional cohort (7.3%; n = 21).

### Factors associated with non-adherence to self-administered systemic therapies

Factors influencing non-adherence may be different for ustekinumab (e.g., delay in administration) compared with self-administered systemic therapies. We did not have data on these “system” factors and for these reasons patients prescribed ustekinumab (n = 160) and those with missing biologic treatment type (n = 34) were excluded from the analyses examining factors associated with adherence. The results from the regression analyses are presented in Table 3.

**Table 3:** The univariable and multivariable regression models for non-adherence to self-administered systemic therapies^1^

Variable	Overall Non-Adherent		Intentionally Non-Adherent		Unintentionally Non-Adherent	
	Univariable	Multivariable	Univariable	Multivariable	Univariable	Multivariable
Demographic						
Age, y	**0.97 (0.96−0.99)**	**0.97 (0.95−0.99)**	1.00 (0.98−1.02)	1.00 (0.97−1.03)	**0.95 (0.93−0.97)**	**0.95 (0.92−0.98)**
n	553	449	553	449	559	451
Male	1.32 (0.87−2.0)	1.67 (0.97−2.89)	1.11 (0.66−1.88)	1.39 (0.71−2.72)	1.03 (0.60−1.76)	1.26 (0.63−2.53)
n	553	449	553	449	559	451
Disease						
Disease duration, y	**0.98 (0.97−1.00)**	1.01 (0.98- 1.03)	1.00 (0.98−1.02)	1.01 (0.99−1.04)	0.98 (0.96−1.00)	1.00 (0.97−1.03)
n	551	449	551	449	557	451
PASI at the start of treatment	0.98 (0.95−1.01)	0.97 (0.94−1.01)	0.98 (0.94−1.02)	0.96 (0.91−1.01)	1.01 (0.97−1.05)	1.00 (0.95−1.04)
n	531	449	531	449	537	451
DLQI at the start of treatment	1.01 (0.99−1.04)	1.01 (0.98−1.05)	1.02 (0.99−1.06)	1.03 (0.98−1.08)	1.01 (0.97−1.05)	0.99 (0.95−1.04)
n	525	449	525	449	530	451
Comorbidities						
Presence of ≥1 comorbidity^2^	0.92 (0.61−1.41)	1.35 (0.75−2.41)	0.87 (0.51−1.49)	0.95 (0.46−1.96)	0.98 (0.56−1.72)	1.65 (0.78−3.49)
n	554	449	554	449	560	451
Inflammatory arthritis	0.91 (0.53−1.55)	1.35 (0.66−2.76)	1.21 (0.63−2.32)	1.78 (0.74−4.24)	1.03 (0.52−2.07)	1.60 (0.64−4.02)
n	551	449	551	449	557	451
Treatment						
Treatment duration, mo	1.01 (1.00−1.03)	**1.04 (1.02−1.06)**	1.02 (1.00 - 1.03)	**1.04 (1.01−1.06)**	1.00 (0.99−1.02)	**1.03 (1.01−1.06)**
n	573	449	573	449	579	451
Conventional therapy	**2.11 (1.42−3.12)**	**4.34 (2.38−7.91)**	**1.80 (1.09−2.97)**	**3.21 (1.54−6.67)**	**2.03 (1.20−3.43)**	**4.87 (2.24−10.59)**
n	599	449	599	449	605	451
Psychological distress (HADS)						
Anxiety	1.03 (0.98−1.07)	1.04 (0.95−1.13)	1.02 (0.97−1.08)	1.04 (0.93−1.16)	1.05 (0.99−1.11)	1.01 (0.91−1.13)
n	588	449	588	449	594	451
Depression	1.01 (0.96−1.06)	0.95 (0.86−1.04)	1.00 (0.94−1.06)	0.93 (0.82−1.04)	1.04 (0.98−1.10)	0.99 (0.88−1.12)
n	588	449	588	449	594	451
Illness beliefs and emotional response toward psoriasis (IPQ-R)						
Illness identity (symptoms)	1.04 (0.97−1.11)	0.99 (0.90−1.09)	1.071 (0.99−1.17)	1.06 (0.95−1.19)	0.98 (0.89−1.08)	0.88 (0.77−1.01)
n	579	449	579	449	585	451
Timeline acute/chronic	0.99 (0.72−1.36)	1.04 (0.62−1.72)	0.86 (0.57−1.28)	0.98 (0.52−1.85)	**1.86 (1.13−3.06)**	**2.16 (1.03−4.52)**
n	589	449	589	449	595	451
Timeline cyclical	1.19 (0.90−1.57)	0.94 (0.63−1.39)	1.28 (0.90−1.83)	0.97 (0.60−1.59)	1.06 (0.74−1.53)	0.80 (0.48−1.31)
n	594	449	594	449	600	451
Consequences	0.99 (0.76−1.29)	0.87 (0.50−1.52)	1.01 (0.72−1.43)	0.71 (0.35−1.45)	**1.46 (1.00−2.11)**	1.15 (0.58−2.30)
n	596	449	596	449	602	451
Personal controllability	**1.36 (1.04**−**1.77)**	1.40 (0.95−2.07)	1.07 (0.76−1.50)	1.12 (0.69−1.81)	1.38 (0.97−1.98)	1.5 (0.92−2.45)
n	591	449	591	449	597	451
Coherence (understanding)	0.83 (0.68−1.01)	0.88 (0.65−1.19)	**0.72 (0.56−0.94)**	0.85 (0.59−1.23)	0.92 (0.70−1.20)	0.78 (0.53−1.14)
n	591	449	591	449	597	451
Emotional response towards psoriasis	1.03 (0.82−1.31)	1.18 (0.72−1.92)	1.19 (0.88−1.61)	1.52 (0.80−2.87)	1.12 (0.82−1.54)	0.95 (0.52−1.73)
n	595	449	595	449	601	451
Medication beliefs						
Group 1 (strongly concerned)^3^	**1.69 (1.12−2.56)**	**1.92 (1.09−3.39)**	**2.35 (1.41−3.90)**	**2.27 (1.16−4.47)**	1.14 (0.64−2.0)	1.00 (0.46−2.16)
n	597	449	597	449	603	451
Strength of the patient’s habit, or routine, for using their prescribed systemic therapy						
Habit	**0.94 (0.92−0.97)**	**0.94 (0.91−0.97)**	**0.96 (0.93−0.98)**	**0.95 (0.92−0.98)**	**0.93 (0.90−0.95)**	**0.92 (0.89−0.96)**
n	570	449	570	449	573	451

Abbreviations: CI, confidence interval; HADS, hospital anxiety and depression scale; IPQ-R, Illness Perception Questionnaire- Revised; OR, odds ratio.

1 Self-administered systemic therapies include methotrexate, cyclosporine, acitretin, fumaric acid esters, adalimumab, and etanercept. Boldface indicates *P* ≤ 0.05.

2 Reference category: no comorbidities (excluding psoriatic arthritis); includes any of hypertension, angina, ischemic heart disease, stroke, epilepsy, asthma, chronic obstructive pulmonary disease, peptic ulcer, renal disease, hepatic disease, tuberculosis, demyelinating disease, diabetes, impaired glucose tolerance, depression, non-skin cancer, immunodeficiency syndrome, thyroid disease, other.

3 Reference category: Medication belief Group 2 (side effects and overuse) and Group 3 (treatment resolved).

#### Overall non-adherence

The multivariable model showed that being on a conventional systemic (odds ratio [OR] = 4.34, 95% CI = 2.38−7.91), having strong medication concerns (OR = 1.92; 95% CI = 1.09−3.39), weaker routine/habit for taking their systemic therapy (OR = 0.94; 95% CI = 0.91−0.97), longer treatment duration (OR = 1.04; 95% CI = 1.02−1.06), and younger age (OR = 0.97, 95% CI = 0.95−0.99) were factors associated with being classified as overall non-adherent. Together, these variables were significantly associated with overall non-adherence (χ^2^ [20] = 73.32, *P* ≤ 0.001) and accounted for 23.4% of the variability in the overall non-adherent variable.

#### Intentional non-adherence

Being on a conventional systemic therapy (OR = 3.21, 95% CI = 1.54−6.67), having strong medication concerns (OR = 2.27, 95% CI = 1.16−4.47), weaker routine/habit for taking their systemic therapy (OR = 0.95; 95% CI = 0.92−0.98), and longer treatment duration (OR = 1.04, 95% CI = 1.01−1.06) were also factors associated with intentional non-adherence. The model was significant (χ^2^ [20] = 40.91, *P* = 0.004) and accounted for 17.0% of the variability in the intentionally non-adherent variable.

#### Unintentional non-adherence

Being on a conventional systemic therapy (OR = 4.87, 95% CI = 2.24−10.59), stronger perceptions of psoriasis being a chronic condition (OR = 2.16, 95% CI = 1.03−4.52), weaker routine/habit for taking their systemic therapy (OR = 0.92, 95% CI = 0.89−0.96), younger age (OR = 0.95, 95% CI 0.92−0.98), and longer treatment duration (OR = 1.03, 95% CI = 1.01−1.06) were factors associated with unintentional non-adherence. The model was significant (χ^2^ [20] = 65.92, *P* = ≤ 0.001) and accounted for 26.6% of the variability in the unintentional non-adherent variable.

## Discussion

### Main findings

This is the largest study to assess adherence to conventional systemic and biologic therapies used for psoriasis in a real-world setting to date; 22.4% of patients using self-administered systemic therapies are classified as non-adherent, with a higher proportion of patients using a conventional systemic classified as non-adherent (29.2%) compared to those using etanercept or adalimumab (16.4%). Patients’ medication beliefs are key drivers of intentional non-adherence. Medication-taking routine or habit strength influences intentional and unintentional non-adherence.

### Comparison to other studies

Adherence is known to be problematic across many long-term conditions (Nieuwlaat et al., 2014), with a recent study in rheumatoid arthritis showing that 27% of patients self-report non-adherence at least once within 6 months of starting a biologic therapy (Bluett et al., 2014). Both higher and lower levels of adherence to systemic therapies used for psoriasis have been reported in other studies reflecting differences in study inclusion criteria and size, and the use of non-validated self-report tools to assess adherence (Thorneloe et al., 2013). Previous adherence research has focused predominantly on demographic and clinical predictors, however, these variables show weak or inconsistent relationships with adherence in psoriasis (Thorneloe et al., 2013) and in other long-term conditions (DiMatteo, 2004), a finding consistent with our results. These factors are not readily amenable to change and have limited utility for informing adherence interventions. We have demonstrated that different “modifiable” factors are associated with intentional and unintentional non-adherence.

In other disease groups, the important influences of patients’ medication beliefs on adherence have been demonstrated (Horne et al., 2013). We have shown that a significant proportion of patients express conflicting beliefs about their systemic therapy; strong beliefs in the perceived need and effectiveness of their systemic therapy and strong concerns about its usage. Patients were more likely to make the decision to use their systemic therapies in ways that have not been prescribed, such as using less of it or stopping treatment for a while (intentional non-adherence) if they had unresolved medication concerns. Patient understanding of the causes and triggers of psoriasis and its disease mechanisms can be low (Nelson et al., 2013a) and the current study suggests that this lack of “illness coherence” may contribute to intentional non-adherence. Patients’ understanding of psoriasis will influence their beliefs about systemic therapies (Horne and Weinman, 2002, Nelson et al., 2017), which is likely to account for the fact that it was not associated with intentional non-adherence after controlling for other variables.

Some instances of intentional non-adherence may be influenced by good disease control; patients may decide to strategically alter their treatment regimen if their symptoms have improved. The impact of disease control on medication non-adherence is likely to be mediated by patients’ beliefs about their psoriasis and medication. Patients who perceive good disease control may report reduced perceived need for their treatment and decide to alter their treatment regimen, especially if they also report concerns about its usage. Psychological distress was not associated with non-adherence. Although depression is a known risk factor for non-adherence in long-term conditions (Grenard et al., 2011), we have previously shown that distress can remain high in psoriasis even if the patient is adhering to their treatment (Thorneloe et al., 2017a).

In other disease groups, the strength of the patient’s routine, or habit, for taking their medication has been shown to predict unintentional non-adherence, such as forgetting to use medication (Bolman et al., 2011, Phillips et al., 2013). Habit-based interventions have been shown to be effective in improving medication adherence (Conn et al., 2017). This study found habit strength was associated with intentional and unintentional non-adherence. Although medication beliefs were not associated with unintentional non-adherence, stronger perceptions of psoriasis as a chronic condition with serious consequences were significant contributors. Future work should explore whether unresolved patient concerns about psoriasis and medication are a barrier to developing good medication-taking routines.

In the United Kingdom, patients generally use topical therapies before commencing a conventional therapy (National Institute for Health and Care Excellence, 2012). Many patients express concerns about topical therapies and perceive or experience poor health care support (Nelson et al., 2013a, Nelson et al., 2013b, Thorneloe et al., 2017a). Beliefs based on their earlier experiences can persist (Rottman et al., 2017) and may influence patients’ behavior or lower expectations about subsequent use of conventional therapies. Fewer patients in the conventional cohort were classified as having positive medication beliefs compared with those using a biologic. Differences in beliefs rather than mode of administration may account for the difference in non-adherence between the conventional and biologic cohorts. The vast majority of patients prescribed ustekinumab were classified as adherent, consistent with its high drug survival (Warren et al., 2015). Ustekinumab is predominately nurse-administered in the United Kingdom; although this will help support adherence patients may still hold unresolved medication concerns or worries about their therapy.

### Implications for clinicians

Clinicians need to be aware of the possibility of non-adherence. More than 20% of patients in this study expressed strong beliefs in the personal need for treatment while simultaneously reporting strong concerns about their use of psoriasis-specific treatments, as well about use of medicines in general. Intentional non-adherence was most common in this group. This group may benefit most from interventions targeting key medication beliefs (Petrie et al., 2012, Phillips et al., 2012). The UK National Institute for Health and Care Excellence (2009) Medicines Adherence Guidelines provides recommendations for supporting shared medication decision-making and adherence. Clinicians should talk with the patient to understand their perspective and establish key medication beliefs before prescribing new treatments and when reviewing medicines. This can be achieved by using standardized assessment tools, such as the Beliefs about Medicines Questionnaire (Horne et al., 1999) or by asking the patient what they know, believe, and understand about their medicines, including any specific concerns they may have about their medication. Doing so will provide an opportunity for the clinician to modify incorrect or incomplete beliefs and help support patient treatment decision-making. Unintentional non-adherence needs an interventional approach that focuses on developing planned and automatic routines for medication-taking (Conn and Ruppar, 2017, O’Carroll et al., 2014). Encouraging the patient to discuss how medication usage fits into their daily life and any practical barriers they may face will help to identify potential solutions and develop good medication-taking habits and routines.

### Strengths and limitations of the study

The real-world cohort study design ensures that patients are representative of those receiving treatment in routine clinical practice. However, an inherent limitation to an observational study is the potential for selection bias. Although the overall sample size was large, 10.5% of patients were not matched to BADBIR and were excluded from the regression models. Adherence rates for the separate systemic therapies should be interpreted with some caution due to smaller number of patients in these specific treatment groups and thus, we were unable to explore why adherence varied by specific treatment type. Ustekinumab is predominately nurse-administered in the United Kingdom, however it is possible that some patients were self-administering. Although it is not possible to infer causality from cross-sectional analyses, the use of appropriate theoretical frameworks and validated data collection tools are major strengths. Although medication belief Groups 2 and 3 had different belief profiles, they were combined in adherence analyses due to the small number of cases in Group 3 being classified as non-adherent.

Self-report tools of adherence can be criticized for being influenced by poor patient recall or reporting bias and so they can overestimate adherence. However, they are the only tools able to distinguish intentional and unintentional non-adherence. The validated Medication Adherence Report Scale has been shown to provide a good estimate of adherence in other conditions (Horne and Weinman, 2002, Ohm and Aaronson, 2006). When we use self-report measures of adherence, it is important that we distinguish non-adherence, where the patient themselves decide to use their treatment in ways that have not been prescribed, from clinician-prescribed cessation of treatment, where the clinician may appropriately stop treatment, for example, due to an infection. We appropriately modified the Medication Adherence Report Scale so the items referred to patient treatment decision-making. If this confounder were present, we would not expect a relationship between beliefs and medication-taking habit/routine with non-adherence. Given that we found this association, we are confident that we measured true non-adherence, rather than appropriate clinician-prescribed variation in treatment usage.

### Summary

Significant proportions of patients with psoriasis prescribed self-administered systemic therapies report intentional and unintentional non-adherence with their treatment regimen. After accounting for relevant variables, patients’ medication beliefs were associated with intentional non-adherence, with the strength of the patients’ medication-taking routine associated with unintentional non-adherence. This study emphasizes the need to assess adherence when determining factors influencing treatment response.

## Materials and Methods

Patients attending 35 dermatology clinics across England were recruited into the iMAP study between March 2013 and September 2016. Patients with a diagnosis of psoriasis under the care of a dermatologist, prescribed an oral conventional systemic (methotrexate, cyclosporine, acitretin, fumaric acid esters), and/or a subcutaneous biologic treatment (etanercept, adalimumab, ustekinumab), aged ≥18 years, and enrolled on BADBIR were eligible for inclusion into the study. In total, 811 psoriasis patients were included in the analysis (Supplementary Figure S1 online).

### Measures

Psoriasis patients were instructed to complete a questionnaire independently and anonymously and return to the investigator. It contained the following measures:

1. The Medication Adherence Report Scale (Horne and Weinman, 2002) that assesses the frequency of intentional (6-items) and unintentional (2-items) non-adherent behaviors on a 5-point Likert scale ranging from very often (= 1) to never (= 5), with higher scores indicating higher levels of adherent behavior. It has been modified and used extensively across many disease groups (Horne and Weinman, 2002, Ohm and Aaronson, 2006). All intentional items were modified to begin with the stem “I decided” (e.g., “I decided to alter the dose of my psoriasis injection/tablets”). Patients were classified into overall adherent/non-adherent categories using a score of ≤38/40, and also classified into intentional (≤28/30) and unintentional (≤8/10) categories (Butler et al., 2004, van den Bemt et al., 2009).
2. The strength of the patient’s habit, or routine, for using their prescribed systemic therapy was assessed using the validated Self-Report Habit Index (Verplanken and Orbell, 2003). Each item is scored on a 5-point Likert scale (strongly disagree [= 1] to strongly agree [= 5]) and summed, with scores ranging from 12 to 60, with higher scores indicating stronger habit.
3. Patients’ beliefs about their psoriasis and emotional responses toward their condition were assessed using the validated Revised Illness Perceptions Questionnaire (Moss-Morris et al., 2002). Seven subscales (39 items) measure perceptions of: how long psoriasis will last (timeline acute/chronic/cyclical); consequences associated with the condition (consequences); personal control; treatment control; understanding of their condition (coherence); and emotional response towards psoriasis. Each item was scored on a 5-point Likert scale (strongly disagree [= 1] to strongly agree [= 5]). A mean score was calculated for each subscale, ranging from 1 to 5, with higher scores indicating stronger illness beliefs and more negative emotional response towards psoriasis. Symptoms attributed to psoriasis (“illness identity”) were assessed (17 items), with each yes-rated symptom scored and summed (yes [= 1], no [= 0]), ranging from 0 to 17. In line with the previous research (Fortune et al., 1998), one psoriasis-specific item was included in the timeline cyclical subscale (“If my psoriasis clears it will always come back”) and three additional symptoms were included in the identity scale (“skin flaking,” “burning sensations,” and “itching”).
4. The Beliefs about Medicines Questionnaire (Horne et al., 1999) assesses: patients’ perceived need for their prescribed systemic therapy (6-items specific-necessity); concerns about the potential adverse consequences of taking it (five items specific-concerns); beliefs in the general harmfulness of medication (four items General-Harm) and; beliefs that medications are overused by doctors (four items General-Overuse). The Beliefs about Medicines Questionnaire was appropriately adapted for use in psoriasis (Thorneloe et al., 2017b). Each item is scored on a 5-point scale (“strongly disagree” [= 1] to “strongly agree” [= 5]), with higher scores indicating stronger medication beliefs. A mean score was calculated for each subscale, with scores ranging from 1 to 5.
5. The Hospital Anxiety and Depression Scale (Zigmond and Snaith, 1983) provides an assessment of symptoms of anxiety (seven items) and depression (seven items) among patients with a physical illness. Patients indicate the strength of agreement with each item on a 0- to 3-point scale. Items are summed to create a Hospital Anxiety and Depression Scale anxiety and depression score, both ranging from 0 to 21. A score of ≥8 indicates a possible or probable caseness of anxiety or depression (Bjelland et al., 2002). Permission was granted to use the Hospital Anxiety and Depression Scale.

Once patients were recruited into the iMAP study, their corresponding data from BADBIR were accessed (with written informed patient consent). Demographic (age, sex) and disease data (age of onset, disease duration, comorbidities) recorded at BADBIR registration were extracted. Treatment variables (treatment type and duration) were extracted at times corresponding to the dates when patients completed the iMAP questionnaire. Psoriasis Area and Severity Index and Dermatology Life Quality Index scores were extracted at the time the patient started the therapy; the closest Psoriasis Area and Severity Index and Dermatology Life Quality Index scores recorded within 6 months of the treatment start date (before or after) were extracted.

### Data analysis

Differences in baseline characteristics between the conventional and biologic cohorts were analyzed using *t*-tests and χ^2^ tests (Tables 1 and 2). LPA was used to identify the optimal number of medication belief groups. The Beliefs about Medicines Questionnaire subscales of specific necessity, specific concerns, general overuse, and general harmfulness, and the Revised Illness Perception Questionnaire subscale of treatment control, were included in the LPA analysis. Three participants had missing data for all five variables and were excluded from the analysis. The model fit statistics for LPA models with one through five class solutions were examined (Supplementary Table S1). These were the Bayesian Information Criterion, where smaller values indicate a better fit, and the Vuong-Lo-Mendell-Rubin Likelihood ratio test and the Lo-Mendell-Rubin Adjusted Likelihood ratio test, both of which compare the current model to a model with one less latent class. Entropy provides a measure of the classification quality of the model, with values approaching 1 indicating a good separation of classes.

Three separate logistic multiple regressions were conducted (Table 3). The odds ratios represent the effect of a 1-unit change in the predictor; they are not standardized and their size depends largely on the units used for the predictor variables. Medication belief Groups 2 and 3 were combined, due to the small number of cases in Group 3 classified as non-adherent (≤8 cases). LPA was conducted in M*plus* 7 (Muthén and Muthén, 1998−2007) and all other analyses were conducted in SPSS, version 23 (IBM, Armonk, NY).

Rates of missing data were very low for the Medication Adherence Report Scale, Self-Report Habit Index, Beliefs about Medicines Questionnaire, Revised Illness Perception Questionnaire and Hospital Anxiety and Depression Scale, ranging from 0.9% to 5.3%. A small number of patients were unable to be matched with data drawn from the registry (Supplementary Figure S1), the amount of missing data was higher for variables obtained from BADBIR, ranging from 6% to 15.7%.

### Ethical approval

Ethical approval for iMAP was obtained from NHS Research Ethics Committee North West England (reference 12/NW/0619) in December 2012 (and from research ethics committees local to each recruiting site). All subjects gave written informed patient consent prior to data collection.

## Conflict of Interest

CEMG has received honoraria and/or research grants from AbbVie, Celgene, LEO Pharma, Lilly, GSK, Janssen, MSD, Novartis, Pfizer, Sandoz, and UCB Pharma.

DMA has received grant funding from AbbVie and the Leo Foundation and served on advisory boards for Pfizer and GSK. LC has received honoraria from Janssen and AbbVie for educational events and an unrestricted research award as a co-applicant from Pfizer. RJT has received an honorarium from Novartis. None of these awards are associated with the submitted manuscript.
