# Supplementary material for: Intentional and Unintentional Medication Non-Adherence in Psoriasis: The Role of Patients’ Medication Beliefs and Habit Strength
Source: J Invest Dermatol. 2018 Apr;138(4):785–94. doi: 10.1016/j.jid.2017.11.015 (PMC5869950; doi:10.1016/j.jid.2017.11.015)

SUPPLEMENTARY MATERIAL

Table S1. Model fit statistics (*n* = 808)

	**Classes**				
	1	2	3	4	5
**Parameters**	10	16	22	28	34
**LL**	-4072.81	-3898.51	-3827.44	-3813.86	-3799.60
**BIC**	8212.56	7904.14	7802.16	7815.17	7826.82
**Entropy**	-	0.60	0.74	0.63	0.66
**VLMR-LRT *p* value**	-	0.00	0.00	0.20	0.17
**LMR-LRT *p* value**	-	0.00	0.00	0.21	0.17

Abbreviations: LL, Log-likelihood; BIC, Bayesian Information Criterion; VLMR-LRT, Vuong-Lo-Mendell-Rubin Likelihood ratio test; LMR-LRT, Lo-Mendell-Rubin Adjusted Likelihood ratio test.

Footnote: Three participants had missing data for all five variables and were excluded from the analysis.

Figure S1. Recruitment flowchart

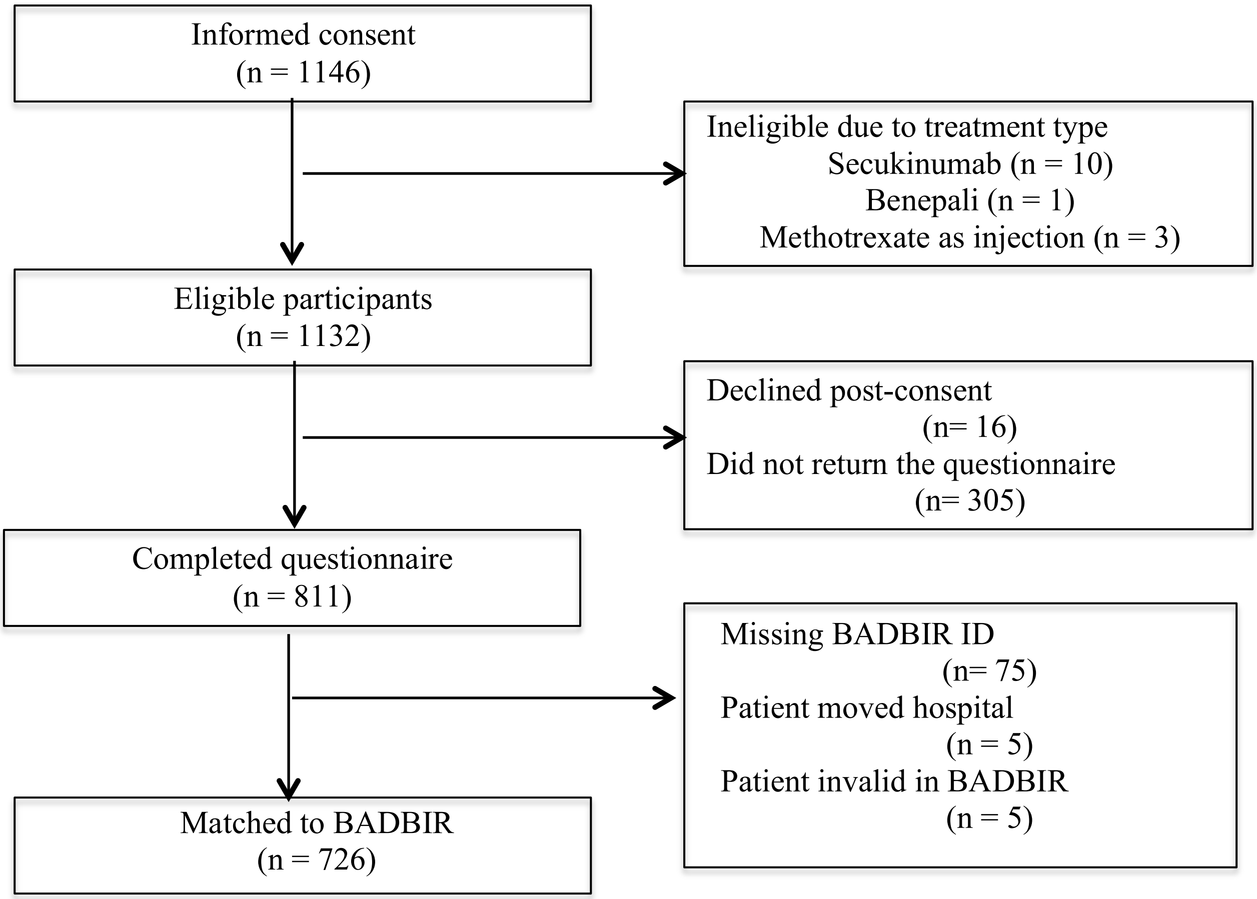

Supplement: Supplementary Table S1 and Supplementary Figure S1 [file mmc1.pdf]
